# Complement Factor H Modulates Splenic B Cell Development and Limits Autoantibody Production

**DOI:** 10.3389/fimmu.2019.01607

**Published:** 2019-07-11

**Authors:** Máté G. Kiss, Mária Ozsvár-Kozma, Florentina Porsch, Laura Göderle, Nikolina Papac-Miličević, Barbara Bartolini-Gritti, Dimitrios Tsiantoulas, Matthew C. Pickering, Christoph J. Binder

**Affiliations:** ^1^Department for Laboratory Medicine, Medical University of Vienna, Vienna, Austria; ^2^CeMM Research Center for Molecular Medicine of the Austrian Academy of Sciences, Vienna, Austria; ^3^Centre for Inflammatory Disease, Imperial College, London, United Kingdom

**Keywords:** complement factor H, complement, autoimmunity, B cell development, B cell receptor signaling

## Abstract

Complement factor H (CFH) has a pivotal role in regulating alternative complement activation through its ability to inhibit the cleavage of the central complement component C3, which links innate and humoral immunity. However, insights into the role of CFH in B cell biology are limited. Here, we demonstrate that deficiency of CFH in mice leads to altered splenic B cell development characterized by the accumulation of marginal zone (MZ) B cells. Furthermore, B cells in *Cf*h^−/−^ mice exhibit enhanced B cell receptor (BCR) signaling as evaluated by increased levels of phosphorylated Bruton's tyrosine kinase (pBTK) and phosphorylated spleen tyrosine kinase (pSYK). We show that enhanced BCR activation is associated with uncontrolled C3 consumption in the spleen and elevated complement receptor 2 (CR2, also known as CD21) levels on the surface of mature splenic B cells. Moreover, aged *Cf*h^−/−^ mice developed splenomegaly with distorted spleen architecture and spontaneous B cell-dependent autoimmunity characterized by germinal center hyperactivity and a marked increase in anti-double stranded DNA (dsDNA) antibodies. Taken together, our data indicate that CFH, through its function as a complement repressor, acts as a negative regulator of BCR signaling and limits autoimmunity.

## Introduction

The complement system is a multiprotein cascade of innate immunity that is specialized in facilitating the clearance of invading pathogens and unwanted host material (1, 2). Furthermore, it has been long appreciated that besides its role in inflammation, complement also participates in regulating adaptive immunity (3), especially through the modulation of B cell responses (4, 5).

Given the complexity of its catalytic nature, the complement cascade is tightly controlled by a wide range of repressors which serve as checkpoints in complement activation both on the cellular surface as well as in the fluid phase (6). Uncontrolled complement activation resulting from defective regulation leads to the accumulation of cellular waste which does not only promote proinflammatory and cytolytic effects inducing host tissue damage but also contributes to the rise of autoimmune conditions as it is seen upon either C1q (7), C4 (8, 9), or CR2/CD21 (10–12) deficiencies in mice as well as in humans (13). Although the role of the classical complement has been well-studied in B cell biology, little is known about how regulators of the alternative pathway affect B cell-mediated immunity.

Complement factor H (CFH) is the master regulator of alternative complement activation. It accelerates the decay of the C3 convertase C3bBb thereby inhibiting the proteolytic cleavage of C3 (14). As one of the most abundant proteins in both human and rodent plasma (100–500 μg/ml), CFH exerts its complement-regulatory effects promptly in the fluid phase. Moreover, it regulates complement on cell surfaces by promoting Factor I-mediated inactivation of C3b into anti-inflammatory iC3b (15). Mice deficient in CFH with no detectable plasma CFH exhibit secondary C3 deficiency due to the constant consumption of circulating C3. Consequently, they develop spontaneous membranoproliferative glomerulonephritis (MPGN) with extensive complement and immune complex deposition on glomeruli (16). Accordingly, renal diseases have been reported in 28 out of 33 individuals with inherited complete deficiency of CFH (17). The pathologic effect has been shown to be dependent on the dysregulation of the alternative complement pathway as additional deletion of complement factor B (FB), which prevents C3 turnover, rescues the phenotype of CFH deficiency.

A recent study that aimed to investigate the contribution of humoral immunity to the development of glomerulopathy employed CFH deficiency as a model for MPGN and showed that *Cf*h^−/−^ mice lacking B cells (backcrossed on the B cell-deficient μ*MT* background) were protected from progressive glomerular disease (18). The authors proposed that antibodies with specificity to certain neo-epitopes exposed on damaged glomeruli exacerbate disease progression, in part by inducing local complement activation. However, it still remains unknown whether the protective effect of B cell deficiency in this model is indirectly linked to CFH deficiency or whether CFH has the capacity to attenuate autoimmunity by directly modulating B cell responsiveness.

Therefore, we characterized B cell immunity in *Cf*h^−/−^ mice and found that in the absence of CFH, splenic B cells exhibit enhanced BCR signaling accompanied by abnormal B cell development. Loss of CFH leads to uncontrolled systemic complement activation which results in increased exposure to activated C3 fragments in the spleen associated with elevated surface levels of CD21 on mature B cells. Aged *Cf*h^−/−^ mice spontaneously develop autoimmunity as judged by germinal center hyperactivity and robust titers of dsDNA-specific immunoglobulins. Our findings provide a novel role for CFH in directly calibrating B cell responsiveness and limiting autoimmunity.

## Materials and Methods

### Mice

*Cf*h^−/−^ mice on a C57BL/6 background were generated by Pickering et al. (16) and were bred in our in-house breeding facility. All experiments were performed with age- and sex-matched mice. Experiments were performed with mice between 9 and 11 weeks of age or 28 and 30 weeks of age, where stated. For the bone marrow transplantation study, 8 week-old C57BL/6 mice were lethally irradiated (2 ×6Gy) and were transplanted with 3 × 10^6^ *Cf*h^+/+^ or *Cf*h^−/−^ bone marrow from 6 week-old donors. The recipient mice were sacrificed after 10 weeks of recovery period. Successful bone marrow reconstitution was verified by extracting and amplifying genomic DNA from the bone marrow of the recipient mice (Supplementary Figure 9). All experimental studies were approved by the Animal Ethics Committee of the Medical University of Vienna, Austria and were performed according to the guidelines for Good Scientific Practice of the Medical University of Vienna, Austria.

### Primary Cell Isolation and Flow Cytometry

Spleens were mechanically dissociated through a 100 μm cell strainer (BD Biosciences) and red blood cells were lysed in red blood cell lysis buffer (Morphisto). Peripheral blood was collected via the vena cava and red blood cells were lysed in red blood cell lysis buffer. Bone marrow cell suspensions were isolated by flushing femurs and tibiae through a 26-gauge needle with 1% FCS in DPBS (Sigma) and red blood cells were lysed as stated above. Total viable cells were counted manually using a hemocytometer or by CASY cell counter & analyzer. For flow cytometric staining, 1 × 10^6^ cells were added in a 96-well V-bottom plate (Thermo Scientific) and incubated with 2.5 μg/ml of a blocking anti-CD16/32 antibody (Clone 90, eBioscience) or anti-CD16/32 APC (Clone 90, eBioscience) diluted in DPBS (Sigma) supplemented with 1% FCS for 20 min at 4°C. After two washing steps, cells were stained with the following monoclonal antibodies: anti-CD45R (B220) PerCP-Cy5.5 (clone RA3-6B2, eBioscience), anti-CD43 PE (clone S7, BD Biosciences), anti-CD23 eFluor450 (clone B3B4, BD Biosciences), anti-CD21/35 BV605 (clone 7G6, BD Biosciences), anti-IgM APC (clone II/41, eBioscience) anti-IgD PE-Cy7 (clone 11-26C, eBioscience), anti-CD11b AlexaFluor700 (clone M1/70, eBioscience), anti-Ly6C BV605 (clone HK1.4, BioLegend), anti-.Ly6G PE (clone 1A8, BioLegend), anti-F4/80 PerCP-Cy5.5 (clone BM8, BioLegend), anti-CD11c APC-eFluor780 (clone N418, eBioscience), anti-CD19 APC (clone eBio1D3, eBioscience), anti-Igκ FITC (clone 197.1, BD Biosciences), biotinylated anti-Igλ (clone RML-42, BioLegend), biotinylated anti-CD138 (clone 281-2, BioLegend), anti-GL7 eFluor450 (clone GL-7, eBioscience), biotinylated anti-CD21/35 (clone 7E9, BioLegend), anti-CD3e PE (clone 145-2C11, eBioscience), anti-CD4 FITC (clone GK1.5, eBioscience), anti-CD8a APC (clone 53-6.7; eBioscience), anti-CD185 (CXCR5) APC (clone SPRCL5, BD Biosciences), anti-CD279 (PD-1) (clone J43, eBioscience), mouse hematopoietic lineage antibody cocktail FITC (17A2, eBioscience), anti-CD117 (c-kit) APC-eFluor 780 (clone 2B8, eBioscience), anti-Sca1 (Ly6A/E) PE-Cy7 (clone D7, eBioscience), anti-CD34 eFluor450 (clone RAM34, eBioscience), anti-CD127 (IL7-R) PE (clone A7R34, eBioscience), anti-CD135 (Flt3) APC-eFluor 710 (clone A2F10, eBioscience), anti-C3b/iC3b,/C3c FITC (clone 3/26, Hycult), anti CD22 APC (clone OX-97, BioLegend), anti-Siglec G APC (clone SH2.1, eBioscience), anti MHCII AlexaFluor700 (clone M5/114.15.2, eBioscience), and streptavidin APC-eFluor 780 (eBioscience).

To determine the amount of intracellular Blimp-1, CD21 and of phosphorylated kinases pBtk and pSyk, cells were fixed and permeabilized with fixation and permeabilization solution (eBioscience) for 20 min at 4°C and then stained intracellularly in permeabilization buffer (eBioscience) with the following antibodies: anti-Blimp-1 Alexa Fluor 647 (clone 5E7; BD Biosciences), anti-CD21/35 PerCP-Cy5.5 (clone 7E9, BioLegend), pBTK/ITK (Y551/Y511) APC (clone M4G3LN; eBiosciences) and pSYK (Y348) APC (clone moch1ct, eBiosciences). Specificity of the intracellular staining was confirmed using Alexa Fluor 647-conjugated isotype controls (Supplementary Figure 10) including rat IgG2a kappa Alexa Fluor 647 (clone cBR2a, eBioscience), mouse IgG2b kappa Alexa Fluor 647 (clone MPC-11, BioLegend) and mouse IgG1 kappa Alexa Fluor 647 (clone P3.6.2.8.1, eBioscience). All stainings were carried out in DPBS (Sigma) supplemented with 1% FCS for 30 min at 4°C, followed by two washing steps. Finally, to identify dead cells staining with 7-AAD viability solution (eBiosciences) was performed. Data were acquired on a LSRII Fortessa (BD Biosciences, Billerica, MA, USA) and were analyzed using FlowJo software 10 (Tree Star, Ashland, OR, USA).

### Antibody Measurements

Anti-dsDNA antibodies were measured as following: 96-well Nunc MaxiSorp plates (Thermo Scientific) were irradiated under UV light for 1 h and then coated with calf thymus DNA (5 μg/ml, Invitrogen) in DPBS. After overnight incubation at 4°C, plates were blocked in 1% BSA in DPBS and incubated in a wet chamber for 1 h at room temperature. Plasma samples were added at 1:80 (IgM) or 1:200 (IgG) dilutions and incubated for 2 h at 37°C. For detection, biotinylated rat anti-mouse IgM (1:2000, BD Pharmingen) or HRP-conjugated anti-mouse IgG (1:1000, GE Healthcare) was added and incubated for 1 h at 37°C. For anti-dsDNA IgM measurements, streptavidin-HRP (R&D Systems) was added in a 1:200 dilution for 30 min at 37°C. Samples were developed using TMB substrate solution according to the manufacturer's instructions. The reaction was stopped with 1M H_2_SO_4_ (Honeywell) and the absorbance was measured with a plate reader at 450 nm as the primary wavelength.

Total IgM, IgG1, IgG2b, IgG2c, IgG3, and IgA levels were measured by a chemiluminescence-based sandwich ELISA as described previously (19). IgE levels were determined using a mouse IgE-specific ELISA (BioLegend). MDA-LDL was prepared as described previously (19). Antigen-specific antibody titers were measured by chemiluminescent ELISA as previously described (20).

### C3 Consumption Measurements

To measure extracellular concentrations of complement components, the spleens were mechanically dissociated through a 100 μm cell strainer (BD Biosciences) and spun down at 400 g for 5 min. The supernatant was collected and C3 and C3a levels were determined using a mouse C3 ELISA kit (Abcam) and a mouse C3a ELISA kit (MyBioSource) according to the manufacturers' instructions. As a measure of C3 consumption, C3a/C3 ratio was calculated based on total C3a concentrations divided by the total C3 levels of each individual sample. Total protein content of the samples was quantified using Pearce BCA Protein Assay Kit (Thermo Fisher Scientific).

### Gene Expression Analysis

Total RNA was isolated from mouse spleen tissue using the RNeasy Mini Kit (PeqLab) and 500 ng of total RNA was reversely transcribed using the High Capacity cDNA Reverse Transcription kit (Applied Biosystems). Quantitative real-time PCR was performed using Kapa SYBR Fast Bio-Rad iCycler with ROX dye (Kapa Biosystems) in a CFX96 Real-time System (Bio-Rad Laboratories). All data were normalized to the housekeeping gene Cyclin B1 (CycB1). Values are expressed as the relative expression compared to the control group.

#### Primer Sequences

mm CycB1-forward: 5′-CAGCAAGTTCCATCGTGTCATCA-3′

mm CycB1-reverse: 5′-GGAAGCGCTCACCATAGATGCTC-3′

mm *C3*-forward: 5′-AGAAAGGGATCTGTGTGGCA-3′

mm *C3*-reverse: 5′- GAAGTAGCGATTCTTGGCGG-3′

For *Cd21* expression measurements, untouched B2 cells from *Cf*h^+/+^ and *Cf*h^−/−^ mice were purified with a B cell isolation kit (Miltenyi) and total RNA was isolated as stated above.

#### Primer Sequences

mm *Cd21*-forward: 5′-CCTCTAACTCATTGCCCCGA-3′

mm *Cd21*-reverse: 5′-AGGAAGCCTTGGTAGCAACT-3′

### Tissue Preparation

Spleens and kidneys were fixed in 10% (v/v) normal buffered formalin, then dehydrated via a Tissue Processor (Leica, TP1020) and embedded in paraffin (Leica, EG1150H). Two micrometer sections were cut (Microm HM335E) and stained with a hematoxylin (Mayer's hematoxylin; Applichem, APC2-254766.1611) and eosin (Eosin Y solution; Sigma, HT110132-1L) stain. Kidneys were also stained with periodic acid (3%, Morphisto, 11839.00500) and Schiff's reagent (Carl Roth, X900.1). Images were taken with Axio Imager A1 from Zeiss.

### Phosphorylated CD19 Staining

Spleen sections were stained for phosphorylated CD19 using an anti-CD19 (phospho Y531) antibody (1:200) by Abcam (ab203615) according to the manufacturers' instructions. Rabbit polyclonal IgG-ChIP Grade (ab27478) was used as isotype control. Biotinylated goat anti-rabbit IgG (H+L) by Vector (BA-1000) was used as a secondary antibody, followed by incubation with streptavidin-peroxidase polymer (Sigma-Aldrich, S2438) and detection with Liquid DAB Substrate (DAKO K3466). Hematoxylin (Mayer's hematoxylin; Applichem, APC2-254766.1611) was applied as counterstain. Images were taken with Axio Imager A1 from Zeiss.

### Scoring of Spleen Architecture

Marginal zone morphology was evaluated as described by Birjandi et al. (21). Shortly, four well-separable white pulp areas were selected from each mouse and the architecture of the marginal zone (MZ) was scored based on two main parameters, the interface distortion and the percent of radius involvement per white pulp area on a scale ranging from 0 to 8 (0—most severe distortions, 8—intact). Data shown are the average score of all white pulp areas per each mouse.

### TUNEL Staining

Spleen sections were stained for TUNEL positivity using an *In Situ* Cell Death Detection Kit, TMR Red by Roche (12156792910) according to the manufacturers' instructions. At least three well-oriented white pulp areas were chosen from each mouse and the number of TUNEL^+^ cells in the MZ area was determined. Data shown are the average score of all white pulp areas per each mouse. Images were taken with Axio Imager A1 from Zeiss.

### Statistical Analysis

Statistical analyses were performed using Graph Pad Prism 7.03 for Windows (Graph Pad Software). Experimental groups were compared using two tailed Student's unpaired or paired *t*-test or Mann-Whitney U test as appropriate. Data are presented as mean ± SEM. A *p*-value of <0.05 was considered significant.

## Results

### Complement Factor H Deficiency Results in Abnormal Splenic B2 Cell Development

**Figure 1:**
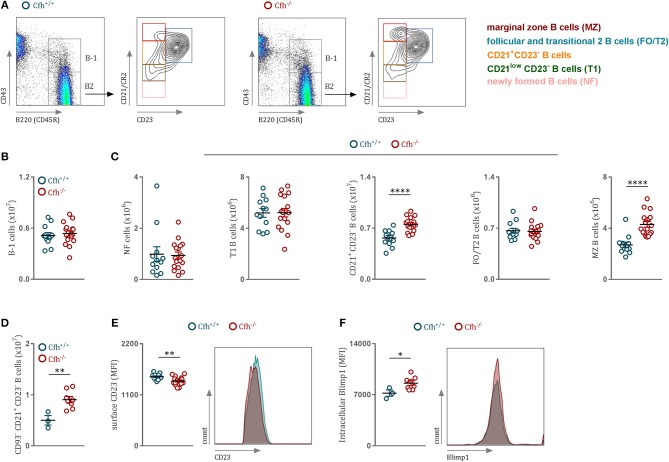
CFH deficiency results in altered splenic B cell development. **(A)** Representative flow cytometry plots showing the gating strategy for B cells in both *Cf*h^+/+^ and *Cf*h^−/−^ mice. **(B)** Absolute numbers of splenic B220^+^CD43^+^ B-1 cells and **(C)** splenic B2 cells in *Cf*h^+/+^ (blue dots) and *Cf*h^−/−^ (red dots) mice assessed by flow cytometry. **(D)** Absolute numbers of splenic B220^+^CD43^−^CD93^−^CD21^+^CD23^−^ B cells of *Cf*h^+/+^ (blue dots) and *Cf*h^−/−^ (red dots) mice analyzed by flow cytometry. **(E)** CD23 mean fluorescence intensity of FO/T2 B cells and **(F)** intracellular Blimp1 mean fluorescence intensity (MFI) of CD21^+^CD23^−^ B cells of *Cf*h^+/+^ (blue dots) and *Cf*h^−/−^ (red dots) mice quantified by flow cytometry. Data shown are pooled from six independent experiments. All results show mean ± SEM, each symbol represents an individual mouse, *p < 0.05, **p < 0.01, ****p < 0.0001 (unpaired *t*-test).

Immunophenotypic characterization of *Cf*h^−/−^ mice revealed that loss of complement factor H leads to markedly elevated counts of splenic CD21^+^ CD23^−^ and marginal zone (MZ) B cells (Figures 1A,C). We found no difference in the numbers of B-1 cells (Figure 1B), newly formed (NF) and transitional stage (T1) B2 cells (Figure 1C) between *Cf*h^+/+^ and *Cf*h^−/−^ mice. Moreover, CFH deficiency did not affect the numbers (Supplementary Figure 1A) or the kappa/lambda light chain ratio (Supplementary Figure 1B) of immature B cells in the bone marrow, suggesting that CFH impacts predominately the splenic B cell developmental process. Consistent with this, exclusion of transitional B220^+^ CD93^+^ B cells had no effect on the increased numbers of CD21^+^ CD23^−^ B cell found in CFH deficient animals (Figure 1D), which suggests an accumulation of mature B2 cells at this developmental stage in the spleen. Although the number of splenic FO/T2 B cells was unchanged in *Cf*h^−/−^ mice (Figure 1C), they exhibited decreased surface expression of CD23 (Figure 1E). Similarly, reduced CD23 levels were found on circulating mature B cells in the blood as well as in the bone marrow (Supplementary Figures 1D,F, respectively), while the numbers were unaffected (Supplementary Figures 1C,E). Blimp1 has been shown to repress CD23 expression in FO B cells (22). In line with this, we detected increased Blimp-1 levels in CD21^+^ CD23^−^ B cells of Cfh^−/−^ mice (Figure 1F). Together, these data indicate that CFH deficiency leads to an accumulation of mature splenic CD21^+^CD23^−^ B cells favoring MZ rather than follicular B cell commitment. Altered splenic B2 cell development in *Cf*h^−/−^ mice had no major effect on total plasma immunoglobulin levels besides a reduction in IgG2c titers (Supplementary Figure 2A) which may be explained by an impaired antigen presentation capacity of splenic B cells due to decreased surface MHCII expression (Supplementary Figure 2B). We also found decreased IgM and IgG antibody levels against MDA-LDL in *Cf*h^−/−^ mice (Supplementary Figure 2C), which can be indicative of increased consumption of MDA-specific immunoglobulins in the absence of the MDA-neutralizing function of CFH (23). In contrast, PC-BSA-specific antibody levels were not different (Supplementary Figure 2D).

### Complement Factor H Deficiency Leads to Increased B Cell Receptor Signaling

**Figure 2:**
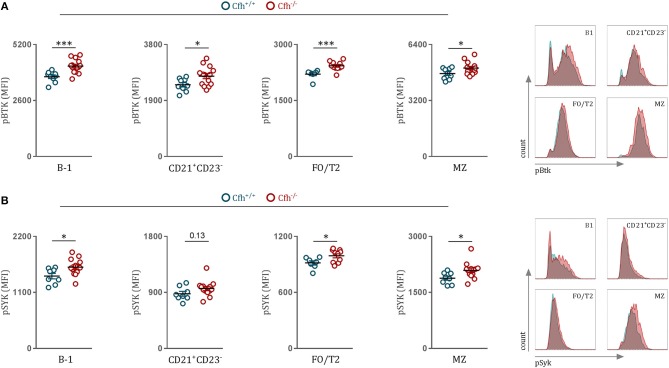
CFH deficiency leads to heightened B cell receptor signaling *in vivo*. **(A,B)** Dot plots and representative flow cytometry plots show the MFI for **(A)** pBTK and **(B)** pSYK levels of B-1, CD21^+^CD23^−^, FO/T2, and MZ B cells (as defined in Figure 1A) of *Cf*h^+/+^ (blue dots) and *Cf*h^−/−^ (red dots) mice. Data shown are pooled from four independent experiments. All results show mean ± SEM, each symbol represents an individual mouse, *p < 0.05, ***p < 0.001 (unpaired *t*-test).

The major driver of B2 cell differentiation toward MZ or FO cell fate is the strength of the BCR signaling (24, 25). B cell receptor (BCR) stimulation leads to the activation of downstream SRC family kinases, such as Bruton's tyrosine kinase (Btk) and spleen tyrosine kinase (Syk), which both have been reported to be crucial players in antigen-receptor signaling and B cell fate decision between MZ and FO B cells (26, 27). Therefore, to study the influence of CFH deficiency on BCR signaling, we chose to quantify phosphorylated Syk and Btk levels of splenic B cell subsets in *Cf*h^+/+^ and *Cf*h^−/−^ mice using a flow cytometry-based approach. First, we confirmed previous findings by our lab and others (24, 28, 29) that strong BCR signaling promotes MZ over FO B cell differentiation, as MZ B cells showed higher phosphorylated Btk levels compared to FO/T2 B cells of *Cf*h^+/+^ mice (FO/T2 B cells, MFI = 2206 ± 113; MZ B cells MFI = 4783 ± 310, *p* < 0.0001). Moreover, we found that both MZ and FO/T2 B cells display elevated levels of phosphorylated Btk and Syk in *Cf*h^−/−^ mice compared to control mice (Figures 2A,B), which could provide an explanation for the accumulation of MZ B cells as a result of CFH deficiency. Increased tyrosine kinase activation was also detected in CD21^+^ CD23^−^ B cells as well as in B-1 cells of *Cf*h^−/−^ mice (Figures 2A,B). Importantly, NF and T1 cells, which require tonic BCR signaling for their survival, showed no difference in pSyk and pBtk levels between *Cf*h^+/+^ and *Cf*h^−/−^ mice (Supplementary Figures 3A,B). Taken together, mature splenic B cells exhibit heightened BCR signaling as a consequence of CFH deficiency which can contribute to the expansion of MZ B cells at the expense of appropriate FO B cell development.

### Increased BCR Signaling Is Associated With Dysregulated Splenic Complement Activation Upon Complement Factor H Deficiency

**Figure 3:**
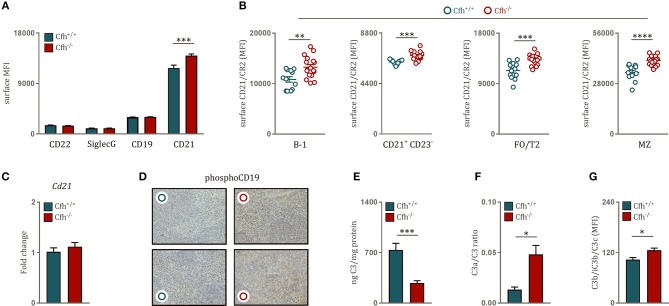
Increased B cell receptor signaling is associated with uncontrolled splenic complement activation upon CFH deficiency. **(A)** Bar graphs showing the MFI of CD22, SiglecG, CD19 and CD21 levels on the surface of B220^+^ B cells of *Cf*h^+/+^ (blue bar) and *Cf*h^−/−^ (red bar) mice measured by flow cytometry. **(B)** Dot plots demonstrating the MFI of surface CD21 levels on B-1, CD21^+^CD23^−^, FO/T2, and MZ B cells (as defined in Figure 1A) of *Cf*h^+/+^ (blue dots) and *Cf*h^−/−^ (red dots) mice analyzed by flow cytometry. **(C)** Relative gene expression of *Cd21* normalized to *CycB1* expression in sorted splenic B2 cells of *Cf*h^+/+^ (blue bar) and *Cf*h^−/−^ (red bar) mice assessed by quantitative PCR. **(D)** Representative images show phosphorylated (Y531) CD19 staining of splenic sections from *Cf*h^+/+^ (blue dots) and *Cf*h^−/−^ (red dots) mice. **(E)** Bar graphs showing total splenic C3 levels of *Cf*h^+/+^ (blue) and *Cf*h^−/−^ (red) mice measured by quantitative C3 ELISA in spleen homogenates. **(F)** C3a/C3 ratio, a measure of complement activation determined by total splenic C3a and C3 levels by quantitative ELISAs. **(G)** Deposition of C3 cleavage products (C3b/iC3b/C3c) on the surface of splenocytes of *Cf*h^+/+^ (blue bar) and *Cf*h^−/−^(red bar) mice analyzed by flow cytometry, shown as MFI. Data shown are pooled from four independent experiments. All results show mean ± SEM, each symbol represents an individual mouse, *p < 0.05, **p < 0.01, ***p < 0.001, ****p < 0.0001 (unpaired *t*-test).

BCR activation is tightly controlled by a variety of co-receptors, which can either inhibit or amplify BCR signaling strength. Therefore, we hypothesized that the effect of CFH deficiency on BCR signaling can be due to altered expression of inhibitory and stimulatory co-receptor signaling. There was no difference in surface levels of the main BCR repressors CD22 and Siglec G on B cells of *Cf*h^+/+^ and *Cf*h^−/−^ mice and similar data were obtained for the positive regulator CD19 (Figure 3A). However, we found significantly higher levels of the co-stimulatory molecule CD21 on the surface of total splenic B cells in *Cf*h^−/−^ mice (Figure 3A), which was found on all mature splenic B cell subsets (Figure 3B). In line with this, mature B cells in the blood as well as in the bone marrow also displayed higher CD21 expression upon CFH deficiency (Supplementary Figures 4A,B). The effect was not due to altered transcriptional regulation as *Cd21* mRNA expression was comparable in sorted splenic B cells from *Cf*h^+/+^ and *Cf*h^−/−^ mice (Figure 3C). In agreement with the latter, splenic B cell subsets of *Cf*h^−/−^ mice showed no difference in intracellular CD21 levels (Supplementary Figure 4C). These findings suggest that increased BCR signaling upon loss of CFH can be in part mediated by enhanced surface CD21 signaling.

CD21 is a member of the B cell co-receptor complex and recognizes active cleavage products of complement component 3 (C3), such as iC3b and C3d (30). C3d acts as a molecular adjuvant in B cell response as interaction of antigen-coupled C3d with CD21 can substantially lower the threshold for BCR activation (31) in a CD19-dependent manner (32). Accordingly, we detected increased phosphorylated CD19 staining in spleen sections of *Cf*h^−/−^ mice compared to littermate controls (Figure 3D). Heightened levels of surface CD21 on splenic B cells can be indicative of complement dysregulation in the spleen. In order to test this, we aimed to characterize local complement activation in the spleen of *Cf*h^+/+^ and *Cf*h^−/−^ mice. We found that spleen of CFH deficient mice contained less extracellular C3 compared to controls (Figure 3E). This was not due to reduced production of C3 by splenocytes (Supplementary Figure 5), but a result of enhanced C3 consumption as demonstrated by a higher C3a/C3 ratio in the spleen of CFH deficient mice (Figure 3F). Consistent with this, increased levels of activated C3b/iC3b/C3c fragments could be detected on the surface of splenocytes derived from *Cf*h^−/−^ mice (Figure 3G). These data show that absence of CFH leads to uncontrolled complement activation in the spleen.

In order to further elucidate whether the influence of CFH on B cell activation is dependent on its systemic complement regulatory function and is not due to a B cell intrinsic effect, we performed a bone marrow transplantation study in which we transplanted lethally irradiated C57BL/6 mice with *Cf*h^+/+^ or *Cf*h^−/−^ bone marrow. Loss of hematopoietic CFH did not result in systemic complement activation as indicated by comparable plasma C3 and C3a levels (Supplementary Figures 6A,B). Moreover, hematopoietic CFH deficiency did not lead to an expansion of MZ B cells (Supplementary Figure 6C) and had no effect on surface CD21/CR2 levels of mature B cells (Supplementary Figure 6D). Consistent with this, intracellular levels of phosphorylated Btk and Syk were unchanged between the two groups (Supplementary Figures 6E,F). These data provide strong evidence, that CFH modulates splenic B cell activation and maturation through its systemic complement regulatory activity.

### Complement Factor H Deficient Mice Develop Germinal Center Hyperactivity With Robust Autoantibody Titers

**Figure 4:**
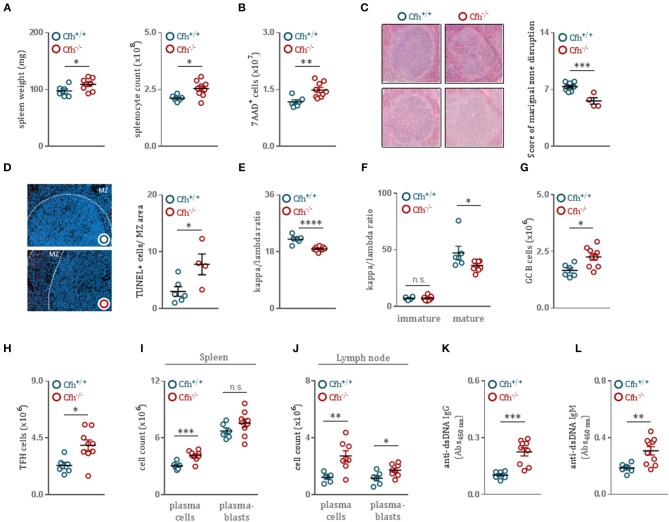
CFH deficient mice develop germinal center hyperactivity with robust autoantibody titers. **(A)** Spleen weight and absolute splenocyte count of 8 month-old *Cf*h^+/+^ (blue dots) and *Cf*h^−/−^ (red dots) mice. **(B)** Total numbers of 7AAD^+^ dying splenocytes of *Cf*h^+/+^ (blue dots) and *Cf*h^−/−^ (red dots) mice, measured by flow cytometry. **(C)** Representative images demonstrating the white pulp architecture of 8 month-old *Cf*h^+/+^ and *Cf*h^−/−^ mice. Dot plots show the score of marginal zone disruption in the spleens of *Cf*h^+/+^ (blue dots) and *Cf*h^−/−^ (red dots) mice. **(D)** Representative images showing TUNEL staining of spleen sections from 8 month-old *Cf*h^+/+^ and *Cf*h^−/−^ mice. Dot plots indicate the average numbers of TUNEL^+^ cells in the marginal zone area in the spleens of *Cf*h^+/+^ (blue dots) and *Cf*h^−/−^ (red dots) mice. Dot plots represent the kappa/lambda light chain ratio of **(E)** IgD^+^CD19^+^CD43^−^ mature splenic B cells as well as **(F)** B220^hi^CD23^+^CD43^−^ mature and B220^lo^CD23^−^CD43^−^ immature splenic B cells of *Cf*h^+/+^ (blue dots) and *Cf*h^−/−^ (red dots) mice assessed by flow cytometry. Absolute numbers of (G) GL-7^+^B220^+^ germinal center B cells and **(H)** CD3^+^CD4^+^CXCR5^+^PD-1^+^ follicular helper T cells in the spleen of *Cf*h^+/+^ (blue dots) and *Cf*h^−/−^ (red dots) mice quantified by flow cytometry. **(I,J)** Quantification of CD138^+^B220^−^ plasma cells and CD138^+^B220^+^ plasmablasts in the spleen as well as in the lymph node of *Cf*h^+/+^ (blue dots) and *Cf*h^−/−^ (red dots) mice by flow cytometry. **(K)** IgG and **(L)** IgM titers specific for double stranded-DNA in the plasma of aged *Cf*h^+/+^ (blue dots) and *Cf*h^−/−^ (red dots) mice determined by ELISA. All results show mean ± SEM, each symbol represents an individual mouse, *p < 0.05, **p < 0.01, ***p < 0.001, ****p < 0.0001 (unpaired *t*-test).

Growing evidence suggests that even modest discrepancies in BCR signaling can promote autoreactivity in the naïve B cell repertoire and predispose to autoimmunity (33). As we found that CFH deficiency results in enhanced B cell activation, we hypothesized that *Cf*h^−/−^ mice develop spontaneous B cell-dependent autoimmune phenotype over time. Indeed, 8 month-old *Cf*h^−/−^ mice presented with splenomegaly (Figure 4A) associated with an increase in dying splenocytes (Figure 4B) compared to age-matched controls. Moreover, CFH deficiency caused severe morphological disruption of the splenic architecture. While spleens of aged *Cf*h^+/+^ mice showed a well-organized white pulp with easily separable marginal zone and lymphoid follicle area, the MZ area of *Cf*h^−/−^ mice were diffuse, poorly discernable, displayed a high degree of distortion (Figure 4C) and contained increased numbers of TUNEL^+^ cells (Figure 4D). The accumulation of dying cells in *Cf*h^−/−^ mice was accompanied by increased splenic inflammatory cell counts including neutrophils, Ly6C^high^ monocytes, Ly6C^low^ monocytes and subsets of macrophages and dendritic cells (Supplementary Figure 7A). This was a consequence of excessive extramedullary hematopoiesis in the spleen (Supplementary Figure 7B) which also led to elevated numbers of inflammatory cells in the periphery (Supplementary Figure 7C).

Importantly, splenic B cells of *Cf*h^−/−^ mice displayed a decreased kappa to lambda chain ratio (Figure 4E and Supplementary Figure 8), suggesting an altered BCR repertoire. Because mature—but not immature—bone marrow B cells of *Cf*h^−/−^ mice showed the same alterations in kappa to lambda ratio as splenic B cells (Figure 4F), we again concluded that the effect is not due to altered BCR editing in the bone marrow, but specific to splenic maturation. The persistence of autoantigens derived from apoptotic material combined with dysregulated BCR signaling can promote germinal center hyperactivity (34). Moreover, autoreactive B cells have the ability to coordinate the expansion of cognate follicular helper T cells to further orchestrate spontaneous germinal center formation. Consistent with this, *Cf*h^−/−^ mice had elevated numbers of germinal center B cells (Figure 4G) as well as follicular helper T cells (Figure 4H) compared to controls. Furthermore, we found increased numbers of plasma cells and plasmablasts both in the spleen (Figure 4I) as well as in the lymph nodes (Figure 4J) of *Cf*h^−/−^ mice, indicative of heightened germinal center activity. In order to test whether the generated plasma cells are autoreactive, we measured anti-dsDNA IgG titers in the plasma of *Cf*h^+/+^ and *Cf*h^−/−^ mice. CFH deficiency resulted in markedly increased levels of dsDNA-specific autoantibodies (Figures 4K,L).

Notably, even in 10 week-old *Cf*h^−/−^ mice increased anti-dsDNA IgG antibodies could be detected (Supplementary Figure 7G) while extramedullary hematopoiesis (Supplementary Figure 7E) as well as splenic and peripheral inflammatory cell counts were unaffected (Supplementary Figures 7D,F) and no overt signs of glomerulonephritis were observed (Supplementary Figure 7H). Thus, CFH deficiency promotes B cell autoimmunity and this precedes chronic inflammation and the development of glomerulonephritis in *Cf*h^−/−^ mice.

## Discussion

CFH deficiency causes uncontrolled complement activation characterized by the continuous cleavage of newly generated C3. Active C3 fragments are crucial mediators of adaptive immune responses. However, whether CFH directly impacts B cells and humoral immunity remains unknown. Here, we demonstrate that CFH deficiency results in heightened BCR signaling which affects splenic B cell development and leads to B cell-dependent autoimmunity with increased levels of dsDNA autoantibodies.

Genetic variants in CFH and CFH-related proteins (CFHRs) have been associated with systemic lupus erythematosus (SLE) development (35). Furthermore, patients with circulating IgG autoantibodies against CFH, which have been shown to inactivate the repressor function of CFH, have increased serum anti-nuclear antibody (ANA) titers (36) and anti-CFH autoantibodies are found in patients positive for lupus anticoagulants (37). Additionally, experimental evidence showed that *Cfh*^−/−^ mice backcrossed on the lupus-prone MRL-lpr background develop accelerated lupus nephritis (LN) which could recapitulate many symptoms of human LN including marked albuminuria and azotemia and resulted in increased mortality at 14 weeks of age (38). However, the exact mechanism behind the development of nephropathies upon CFH deficiency has not yet been addressed. Recently, evidence for a B cell-mediated pathological effect of CFH deficiency came from a study, in which *Cf*h^−/−^ mice were crossed with mice lacking functional B cells (18). While aged *Cf*h^−/−^ mice developed spontaneous renal disease with omnipresent deposition of complement components on the glomeruli, CFH deficient mice lacking B cells were protected from renal damage. In accordance with this, our data provide a direct link between CFH and B-cell dependent autoimmunity, as we found that CFH controls splenic C3 cleavage and modulates BCR signaling, thus contributing to physiological B cell development.

The strength of BCR signaling is the major determinant of the developmental fate of mature splenic B cells toward MZ or FO B cell maturation (25). Initially, BCR signaling strength was thought to be directly associated with FO B cell differentiation (39, 40). We and others have provided direct evidence that strong BCR signaling favors MZ B cell over FO cell development (24, 28, 29). Mice deficient in secreted IgM displayed increased MZ B cell and decreased FO B cell numbers and low-dose treatment of *sIg*M^−/−^ mice with the Btk inhibitor Ibrutinib promoted differentiation of FO B cells while restricting MZ B cell formation. This is in accordance with our current report that enhanced BCR signaling in CFH deficient mice leads to the doubling of the MZ B cell pool, although other potential mechanisms independent of BCR signaling may also contribute to increased MZ B cell numbers. It is important to note that similar to mice with CFH deficiency, *sIg*M^−/−^ mice also develop high autoantibody titers and autoimmunity (41). The protective effect of secreted IgM is attributed to its ability to dampen self-antigen-induced BCR signaling (24). Sources of such self-antigens include cell debris, dying cells and microvesicles (42, 43). Similarly, CFH is able to bind immunogenic ligands such as MDA (23), DNA, histones and annexin-II (44) on the surface of apoptotic and necrotic cells and mediate their anti-inflammatory disposal. The importance of cell surface recognition by CFH is well-illustrated in a study showing that mice with a mutant CFH that lack its surface recognition domains (including an MDA-binding site) develop spontaneous kidney disease (45). While in this study we did not investigate whether CFH could neutralize MDA-induced stimulatory effects on B cell activation, we found that CFH deficient mice show decreased plasma levels of antibodies specific to MDA epitopes, likely due to increased consumption of MDA-reactive antibodies. Therefore, it is plausible that CFH might control B cell activation in part by sequestering self-antigens.

B cell activation is tightly modulated by accessory transmembrane molecules in the close proximity of the BCR (46). CD21 is part of the stimulatory BCR co-receptor complex and its differential expression defines the developmental stage of splenic B cells. It has been suggested that complement factor H-related protein 3 (CFHR3) binds C3d and can abrogate its interaction with CD21 thereby preventing the interaction of the BCR with the co-receptor complex on human B cells (47). Here, we identify CFH as a crucial modulator of surface CD21 expression on mature B cells in mice. While CD21 levels and the BCR signaling strength were comparable in immature B cells of *Cf*h^+/+^ and *Cf*h^−/−^ mice, we detected increased CD21 expression on the surface of mature B cells in the absence of CFH concurrent with heightened BCR signaling. CD21 binds active C3 cleavage products and we show that uncontrolled systemic C3 activation as a consequence of CFH deficiency also results in increased C3 activation in the spleen. Therefore, we propose that increased exposure to C3d-coated antigens initiates an amplification loop in B cell activation, which leads to increased CD21 surface levels further enhancing BCR signaling.

Inadequate BCR signaling can culminate in the escape and activation of autoreactive B cells by facilitating the formation of autoimmune germinal centers in lymphoid follicles (33, 48). Accordingly, autoantibody-secreting B cells derived from patients with SLE often display somatic hypermutation (49). Consistent with this, we found that CFH deficiency results in germinal center hyperactivity, elevated numbers of germinal center B cells and follicular helper T cells and an altered BCR repertoire. High levels of pathogenic autoantibodies secreted by autoreactive plasma cells are a hallmark of autoimmunity. Here we demonstrate that *Cf*h^−/−^ mice have increased titers of anti-dsDNA immunoglobulins and develop systemic autoimmunity over time. In our hands, the presence of autoantibodies preceded the progression of glomerulonephritis as we could detect anti-dsDNA IgGs upon CFH deficiency as early as at 10 weeks of age while no apparent renal pathology could be seen between *Cf*h^+/+^ and *Cf*h^−/−^ mice at this age. The occurrence of autoantibodies was concurrent with heightened BCR signaling in *Cf*h^−/−^ mice, thereby implicating the loss of B cell tolerance in the development of autoantibodies upon CFH deficiency. This is supported by studies showing that transgenic mice overexpressing Btk have increased circulating ANAs (50, 51) and develop a lupus-like disease. Therefore, it is tempting to speculate that treatment of *Cf*h^−/−^ mice with Ibrutinib would reverse the adverse effects of CFH deficiency on germinal center hyperactivity and autoantibody production (52, 53).

In conclusion, we found that upon aging, *Cf*h^−/−^ mice develop high titers of anti-dsDNA IgG antibodies and suffer from autoimmunity joint with chronic inflammation as a consequence of excessive hematopoiesis in the spleen. In the absence of CFH, uncontrolled complement activation results in heightened BCR signaling, altered splenic B cell development and germinal center hyperactivity leading to the expansion of autoreactive plasma cells. Our findings identify a previously undefined role for CFH in protecting from autoimmunity and chronic inflammation.

## Data Availability

This manuscript contains previously unpublished data. The name of the repository and accession number are not available.

## Ethics Statement

This study was carried out in accordance with the recommendations of the guidelines of Good Scientific Practice of the Medical University of Vienna (Austria). The protocol was approved by the Animal Ethics Committee of the Medical University of Vienna (Austria) 66.009/0132-WF/V/3b/2015.

## Author Contributions

MK conceived the project, designed and performed experiments, analyzed and interpreted the data, and wrote the manuscript. MO-K, FP, LG, NP-M, and BB-G performed experiments. DT critically revised and edited the manuscript. MP critically revised the manuscript and provided the *Cf*h^−/−^ mice. CB conceived the project, designed experiments, interpreted the data, and wrote the manuscript.

### Conflict of Interest Statement

The authors declare that the research was conducted in the absence of any commercial or financial relationships that could be construed as a potential conflict of interest.

## Supplementary Material

Supplementary Figure 1 CFH deficiency limits CD23 expression of circulating and bone marrow B cells without affecting their numbers. **(A)** Representative flow cytometry plots showing the gating strategy for B cells and dot plots demonstrating immature bone marrow B cell numbers in *Cf*h^+/+^ (blue dots) and *Cf*h^−/−^ (red dots) mice quantified by flow cytometry. **(B)** Bar graphs representing the kappa/lambda light chain ratio of immature B cells in the bone marrow of *Cf*h^+/+^ (blue bar) and *Cf*h^−/−^ (red bar) mice analyzed by flow cytometry. **(C)** Absolute numbers of mature bone marrow B cells and **(D)** their CD23 expression in *Cf*h^+/+^ (blue dots) and *Cf*h^−/−^ (red dots) mice measured by flow cytometry. **(E)** Representative flow cytometry plots showing the gating strategy for circulating CD23^+^ B cells and dot plots indicating the count and **(F)** CD23 expression of CD23^+^ B cells in *Cf*h^+/+^ (blue dots) and *Cf*h^−/−^ (red dots) mice quantified by flow cytometry. Data shown are pooled from two independent experiments. All results show mean ± SEM, each symbol represents an individual mouse, ^*^*p* < 0.05, ^**^*p* < 0.01 (unpaired *t*-test).

Supplementary Figure 2 Immunoglobulin levels in Cfh^+/+^ and Cfh^−/−^ mice. **(A)** Quantification of total IgM, IgG1, IgG2b, IgG2c, IgG3, IgA, and IgE levels in the plasma of *Cf*h^+/+^ (blue dots) and *Cf*h^−/−^ (red dots) mice measured by quantitative ELISAs. **(B)** Dot plots demonstrate the MFI of surface MHCII levels on FO/T2 and MZ B cells (as defined in Figure 1A) of *Cf*h^+/+^ (blue dots) and *Cf*h^−/−^ (red dots) mice analyzed by flow cytometry. **(C)** MDA-LDL specific antibody levels (IgM, IgG1, IgG2b, IgG2c, and IgG3) and **(D)** PC-BSA specific antibody levels (IgM and IgG) in the plasma of *Cf*h^+/+^ (blue dots) and *Cf*h^−/−^ (red dots) mice determined by ELISA, shown as relative light unit (RLU)/100 ms. All results show mean ± SEM, each symbol represents an individual mouse, ^*^*p* < 0.05, ^**^*p* < 0.01 (unpaired *t*-test).

Supplementary Figure 3 CFH deficiency does not influence BCR signaling of newly formed (NF) and T1 B cells in the spleen. **(A)** Dot plots show the mean fluorescence intensity (MFI) for **(A)** pBTK and **(B)** pSYK levels of NF and T1 B cells (as defined in Figure 1A) in *Cf*h^+/+^ (blue dots) and *Cf*h^−/−^ (red dots) mice. Data shown are pooled from four independent experiments. All results show mean ± SEM (unpaired *t*-test), each symbol represents an individual mouse.

Supplementary Figure 4 Blood and bone marrow B cells display increased CD21 expression upon loss of CFH. **(A)** CD21 mean fluorescence intensity (MFI) of circulating CD23^+^ B cells (as defined in Supplementary Figure 1E) and **(B)** mature bone marrow B cells (as defined in Supplementary Figure 1A) in *Cf*h^+/+^ (blue dots) and *Cf*h^−/−^ (red dots) mice analyzed by flow cytometry. **(C)** Intracellular CD21 levels (MFI) of B-1, CD21^+^CD23^−^, FO/T2 and MZ B cells (as defined in Figure 1A) of *Cf*h^+/+^ (blue dots) and *Cf*h^−/−^ (red dots) mice quantified by flow cytometry. Data shown are representative of three independent experiments. All results show mean ± SEM, ^*^*p* < 0.05, ^**^*p* < 0.01 (unpaired *t*-test), each symbol represents an individual mouse.

Supplementary Figure 5 CFH does not alter splenic C3 production. Relative gene expression of *C3* normalized to *CycB1* expression in the spleen of *Cf*h^+/+^ (blue bar) and *Cf*h^−/−^ (red bar) mice assessed by quantitative PCR. Data shown are pooled from four independent experiments.

Supplementary Figure 6 Hematopoietic deletion of CFH does not affect systemic complement activation, B cell signaling and maturation. **(A,B)** Dot plots showing total plasma **(A)** C3 and **(B)** C3a levels of C57BL/6 mice receiving *Cf*h^+/+^ (blue) or *Cf*h^−/−^ (red) bone marrow measured by quantitative ELISAs. **(C)** Absolute numbers of MZ B cells of C57BL/6 mice receiving *Cf*h^+/+^ (blue) or *Cf*h^−/−^ (red) bone marrow assessed by flow cytometry. **(D)** Dot plots demonstrating the MFI of surface CD21 levels on B-1, CD21^+^CD23^−^, FO/T2 and MZ B cells (as defined in Figure 1A) of C57BL/6 mice receiving *Cf*h^+/+^ (blue) or *Cf*h^−/−^ (red) bone marrow analyzed by flow cytometry. **(E,F)** Dot plots showing the MFI for **(E)** pBTK and **(F)** pSYK levels of B-1, CD21^+^CD23^−^, FO/T2, and MZ B cells (as defined in Figure 1A) of C57BL/6 mice transplanted with *Cf*h^+/+^ (blue) or *Cf*h^−/−^ (red) bone marrow measured by flow cytometry.

Supplementary Figure 7 Autoantibody production precedes chronic inflammation and renal pathology in CFH deficient mice. **(A,D)** Absolute numbers of CD11b^+^Ly6C^+^Ly6G^+^ neutrophils, CD11b^+^Ly6C^hi^Ly6G^−^ monocytes, CD11b^+^Ly6C^lo^Ly6G^−^ monocytes, CD11b^−^F4/80^+^ macrophages, CD11b^+^F4/80^+^ macrophages, CD11b^−^CD11c^+^ dendritic cells, and CD11b^+^ CD11c^+^ dendritic cells in the spleen of **(A)** 8 month-old and **(D)** 10 week-old *Cf*h^+/+^ (blue dots) and *Cf*h^−/−^ (red dots) mice quantified by flow cytometry. **(B,E)** Absolute numbers of Lin^−^c-kit^+^Sca-1^+^ LSKs and Lin^−^c-kit^+^Sca-1^−^CD34^+^CD16/32^hi^ granulocyte-macrophage progenitors (GMP) in the spleen of **(B)** 8 month-old and **(E)** 10 week-old *Cf*h^+/+^ (blue dots) and *Cf*h^−/−^ (red dots) mice by flow cytometry. **(C,F)** Absolute numbers of CD11b^+^Ly6C^+^Ly6G^+^ neutrophils, CD11b^+^Ly6C^hi^Ly6G^−^ monocytes and CD11b^+^Ly6C^lo^Ly6G^−^ monocytes in the peripheral blood of **(C)** 8 month-old and **(F)** 10 week-old *Cf*h^+/+^ (blue dots) and *Cf*h^−/−^ (red dots) mice analyzed by flow cytometry. **(G)** IgG titers specific for double stranded-DNA in the plasma of 10 week-old *Cf*h^+/+^ (blue dots) and *Cf*h^−/−^ (red dots) mice determined by ELISA. Data shown are pooled from four independent experiments. **(H)** Hematoxylin-eosin and Periodic acid-Schiff staining of kidney sections of 10 week-old *Cf*h^+/+^ (blue dots) and *Cf*h^−/−^ (red dots) mice, images are representative of 5 mice per group. All results show mean ± SEM, each symbol represents an individual mouse, ^*^*p* < 0.05, ^**^*p* < 0.01 (unpaired *t*-test).

Supplementary Figure 8 CFH deficiency leads to decreased kappa to lambda chain ratio of both FO/T2 B cells and MZ B cells. Dot plots represent the kappa/lambda light chain ratio of FO/T2 and MZ B cells (as defined in Figure 1A) of *Cf*h^+/+^ (blue dots) and *Cf*h^−/−^ (red dots) mice assessed by flow cytometry. Results show mean ± SEM, each symbol represents an individual mouse, ^*^*p* < 0.05 (unpaired *t*-test).

Supplementary Figure 9 Successful bone marrow reconstitution. At time of sacrifice bone marrow cells were collected from recipient mice reconstituted with *Cf*h^+/+^ and *Cf*h^−/−^ donor cells and genomic DNA was extracted and amplified for the *Cfh* gene. Tail DNA from *Cf*h^+/+^, *Cf*h^+/−^ and *Cf*h^−/−^ mice were used as positive and negative controls.

Supplementary Figure 10 Isotype control staining of intracellular Blimp1, pBTK, and pSYK levels. Histograms show Blimp1 and isotype rat IgG2a kappa staining of B220^+^ B cells as well as pBTK and isotype mouse IgG2b kappa and pSYK and isotype mouse IgG1 kappa staining of B220^+^CD43^−^CD21^hi^CD23^−^ marginal zone B cells of *Cf*h^+/+^ (blue histograms) and *Cf*h^−/−^ (red histograms) mice assessed by flow cytometry.
