# Supplementary figures and images for: Complement Factor H Modulates Splenic B Cell Development and Limits Autoantibody Production
Source: Front Immunol. 2019 Jul 11;10:1607. doi: 10.3389/fimmu.2019.01607 (PMC6637296; doi:10.3389/fimmu.2019.01607)

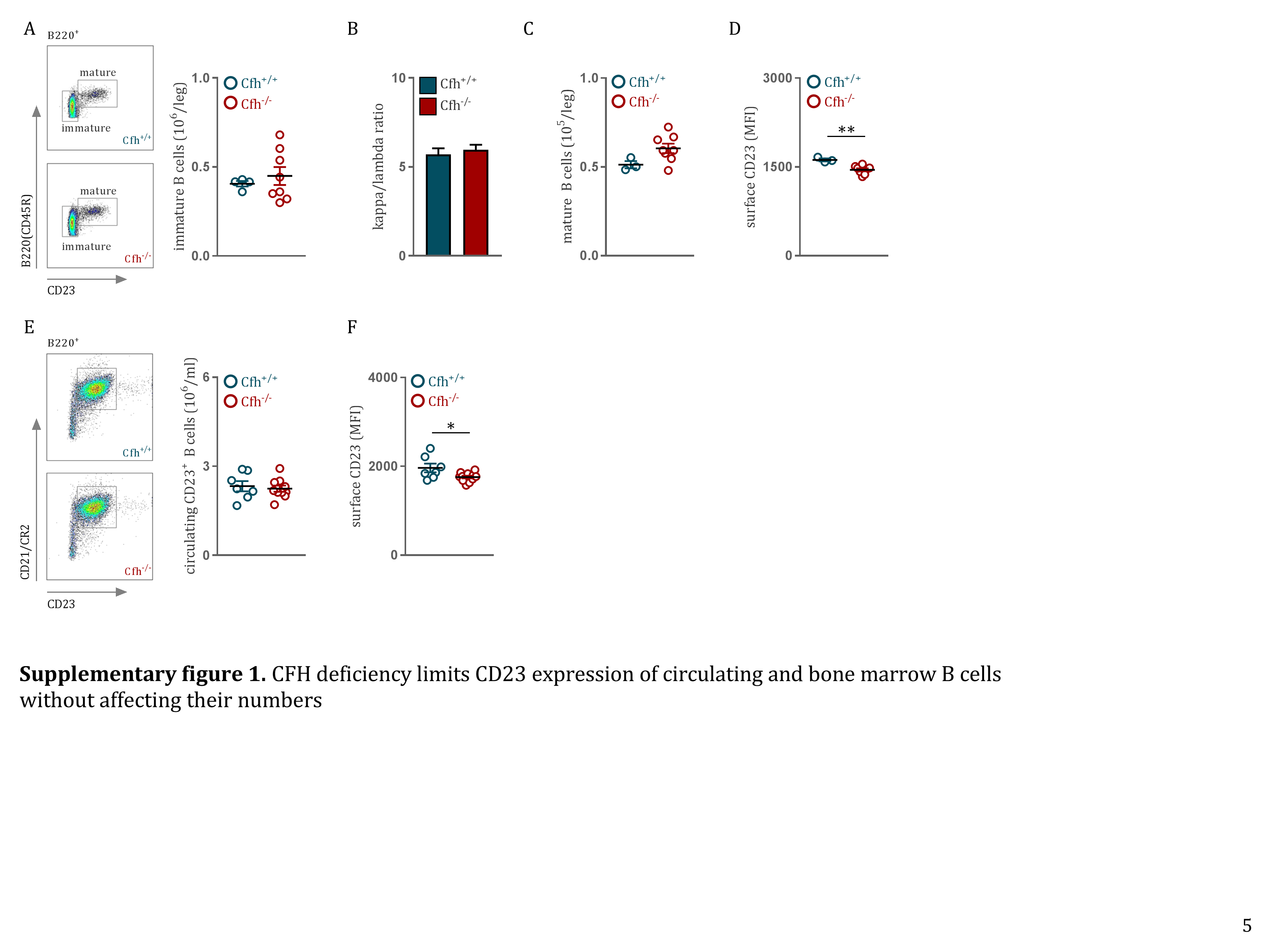

Supplement: Supplementary Figure 1 — CFH deficiency limits CD23 expression of circulating and bone marrow B cells without affecting their numbers. (A) Representative flow cytometry plots showing the gating strategy for B cells and dot plots demonstrating immature bone marrow B cell numbers in Cfh+/+ (blue dots) and Cfh−/− (red dots) mice quantified by flow cytometry. (B) Bar graphs representing the kappa/lambda light chain ratio of immature B cells in the bone marrow of Cfh+/+ (blue bar) and Cfh−/− (red bar) mice analyzed by flow cytometry. (C) Absolute numbers of mature bone marrow B cells and (D) their CD23 expression in Cfh+/+ (blue dots) and Cfh−/− (red dots) mice measured by flow cytometry. (E) Representative flow cytometry plots showing the gating strategy for circulating CD23+ B cells and dot plots indicating the count and (F) CD23 expression of CD23+ B cells in Cfh+/+ (blue dots) and Cfh−/− (red dots) mice quantified by flow cytometry. Data shown are pooled from two independent experiments. All results show mean ± SEM, each symbol represents an individual mouse, *p < 0.05, **p < 0.01 (unpaired t-test). [file Data_Sheet_1.zip › Figure 1.TIF]

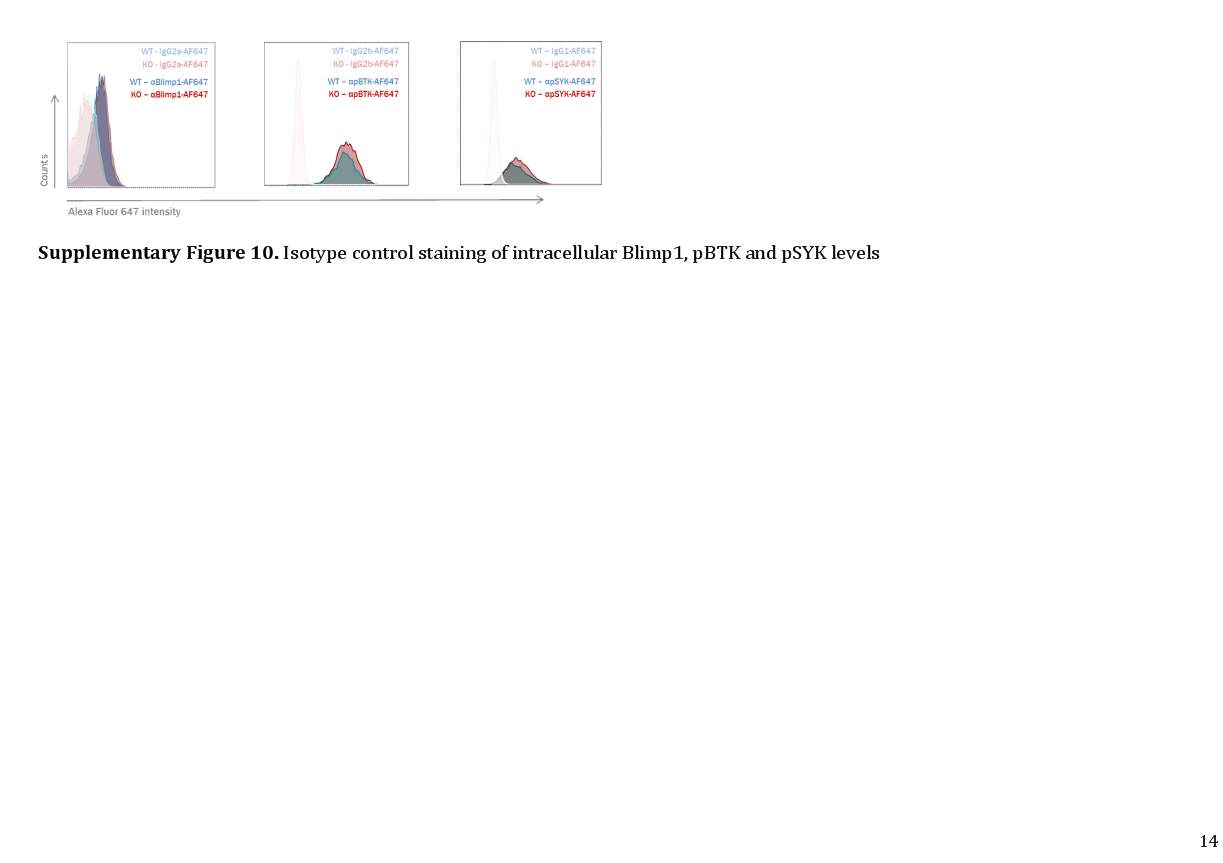

Supplement: Supplementary Figure 1 — CFH deficiency limits CD23 expression of circulating and bone marrow B cells without affecting their numbers. (A) Representative flow cytometry plots showing the gating strategy for B cells and dot plots demonstrating immature bone marrow B cell numbers in Cfh+/+ (blue dots) and Cfh−/− (red dots) mice quantified by flow cytometry. (B) Bar graphs representing the kappa/lambda light chain ratio of immature B cells in the bone marrow of Cfh+/+ (blue bar) and Cfh−/− (red bar) mice analyzed by flow cytometry. (C) Absolute numbers of mature bone marrow B cells and (D) their CD23 expression in Cfh+/+ (blue dots) and Cfh−/− (red dots) mice measured by flow cytometry. (E) Representative flow cytometry plots showing the gating strategy for circulating CD23+ B cells and dot plots indicating the count and (F) CD23 expression of CD23+ B cells in Cfh+/+ (blue dots) and Cfh−/− (red dots) mice quantified by flow cytometry. Data shown are pooled from two independent experiments. All results show mean ± SEM, each symbol represents an individual mouse, *p < 0.05, **p < 0.01 (unpaired t-test). [file Data_Sheet_1.zip › Figure 10.TIF]

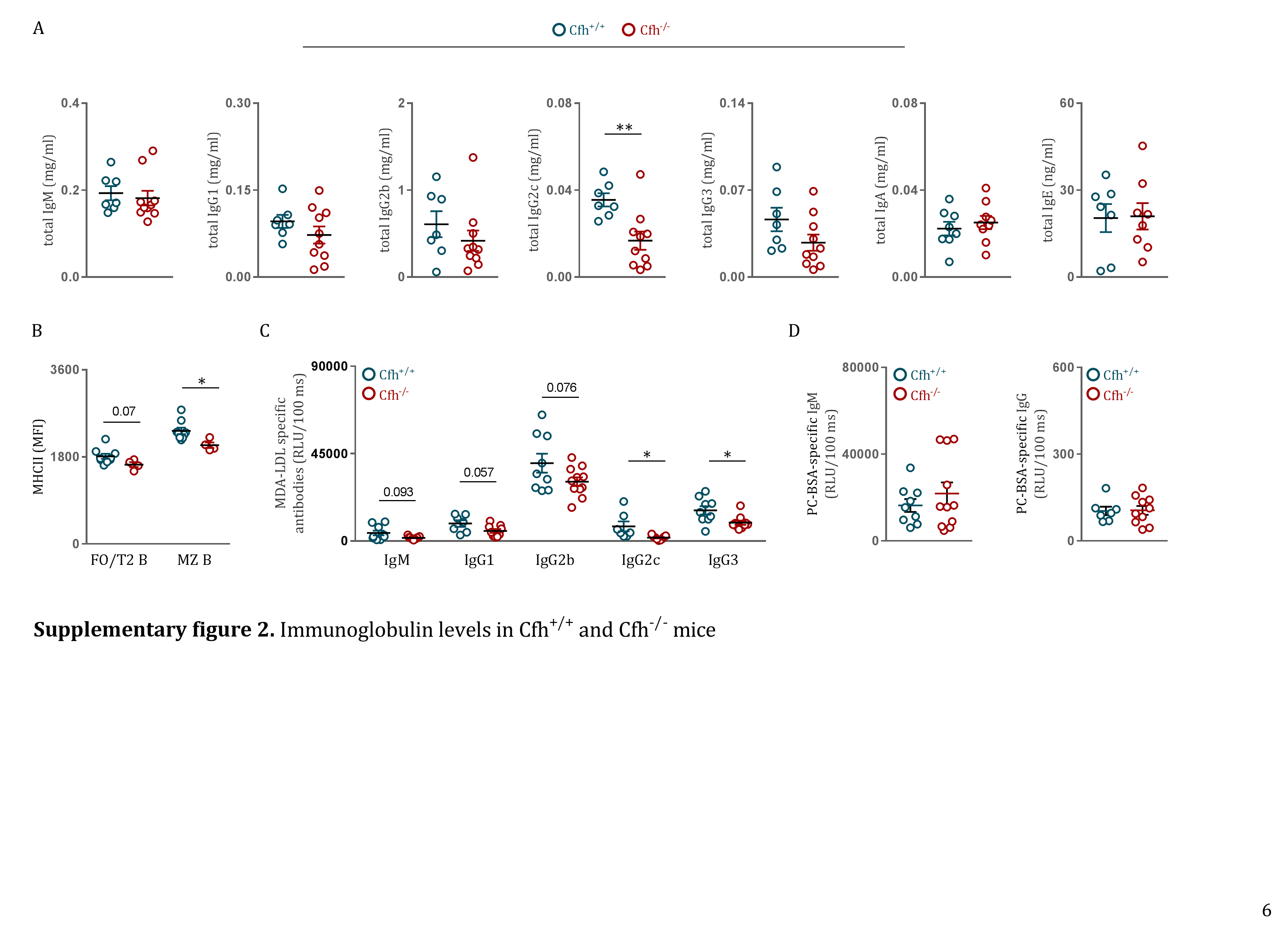

Supplement: Supplementary Figure 1 — CFH deficiency limits CD23 expression of circulating and bone marrow B cells without affecting their numbers. (A) Representative flow cytometry plots showing the gating strategy for B cells and dot plots demonstrating immature bone marrow B cell numbers in Cfh+/+ (blue dots) and Cfh−/− (red dots) mice quantified by flow cytometry. (B) Bar graphs representing the kappa/lambda light chain ratio of immature B cells in the bone marrow of Cfh+/+ (blue bar) and Cfh−/− (red bar) mice analyzed by flow cytometry. (C) Absolute numbers of mature bone marrow B cells and (D) their CD23 expression in Cfh+/+ (blue dots) and Cfh−/− (red dots) mice measured by flow cytometry. (E) Representative flow cytometry plots showing the gating strategy for circulating CD23+ B cells and dot plots indicating the count and (F) CD23 expression of CD23+ B cells in Cfh+/+ (blue dots) and Cfh−/− (red dots) mice quantified by flow cytometry. Data shown are pooled from two independent experiments. All results show mean ± SEM, each symbol represents an individual mouse, *p < 0.05, **p < 0.01 (unpaired t-test). [file Data_Sheet_1.zip › Figure 2.TIF]

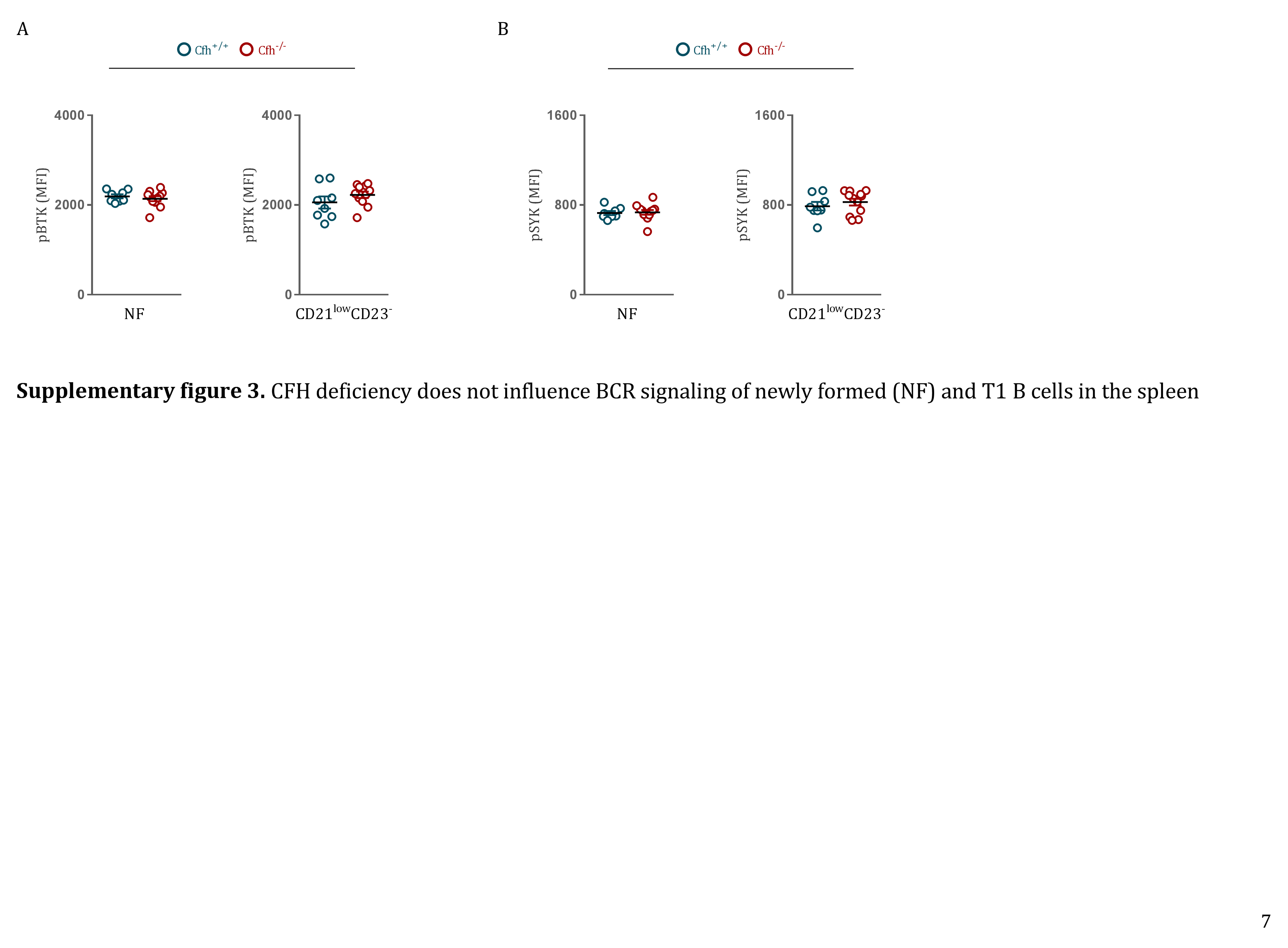

Supplement: Supplementary Figure 1 — CFH deficiency limits CD23 expression of circulating and bone marrow B cells without affecting their numbers. (A) Representative flow cytometry plots showing the gating strategy for B cells and dot plots demonstrating immature bone marrow B cell numbers in Cfh+/+ (blue dots) and Cfh−/− (red dots) mice quantified by flow cytometry. (B) Bar graphs representing the kappa/lambda light chain ratio of immature B cells in the bone marrow of Cfh+/+ (blue bar) and Cfh−/− (red bar) mice analyzed by flow cytometry. (C) Absolute numbers of mature bone marrow B cells and (D) their CD23 expression in Cfh+/+ (blue dots) and Cfh−/− (red dots) mice measured by flow cytometry. (E) Representative flow cytometry plots showing the gating strategy for circulating CD23+ B cells and dot plots indicating the count and (F) CD23 expression of CD23+ B cells in Cfh+/+ (blue dots) and Cfh−/− (red dots) mice quantified by flow cytometry. Data shown are pooled from two independent experiments. All results show mean ± SEM, each symbol represents an individual mouse, *p < 0.05, **p < 0.01 (unpaired t-test). [file Data_Sheet_1.zip › Figure 3.TIF]

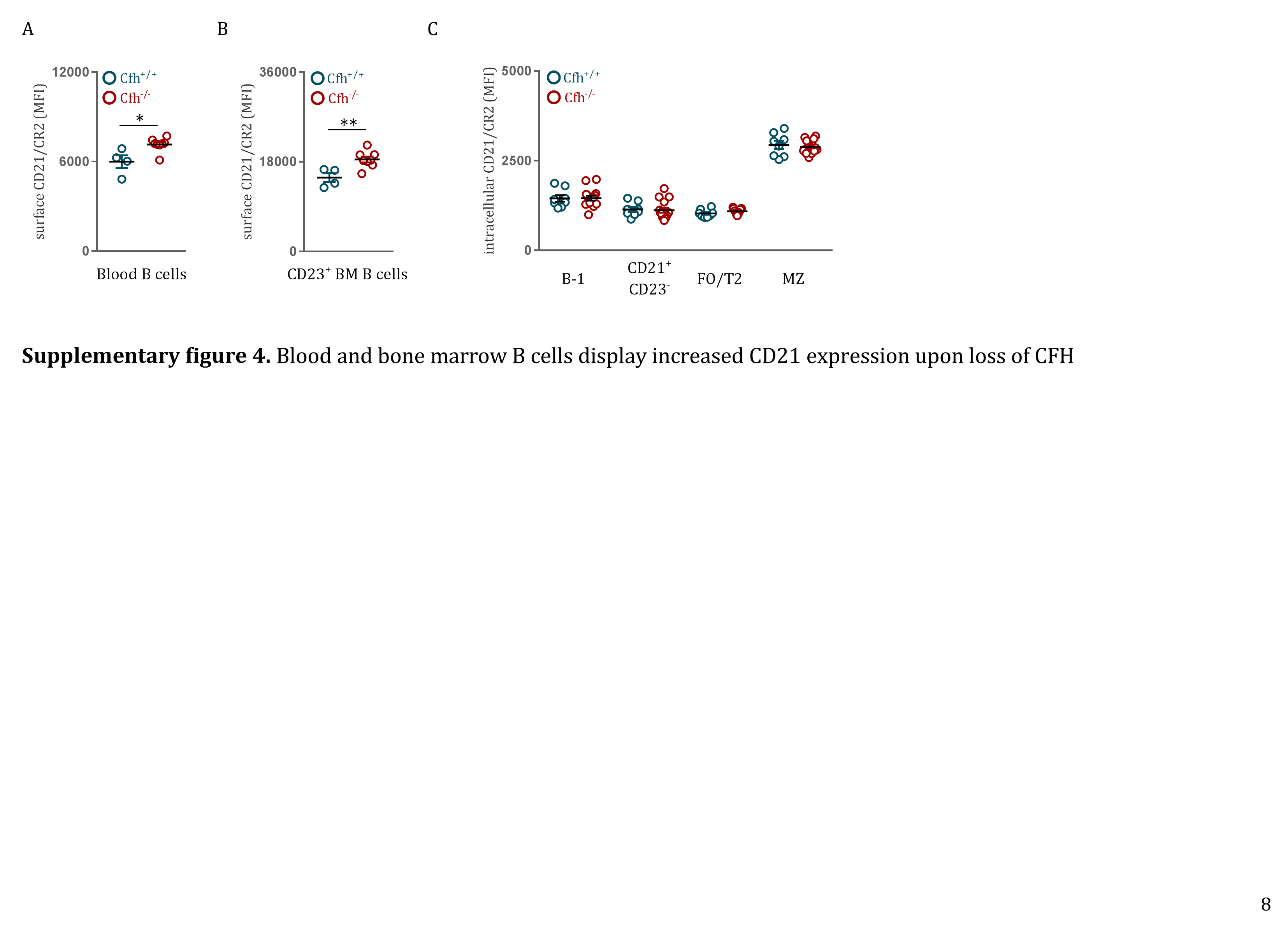

Supplement: Supplementary Figure 1 — CFH deficiency limits CD23 expression of circulating and bone marrow B cells without affecting their numbers. (A) Representative flow cytometry plots showing the gating strategy for B cells and dot plots demonstrating immature bone marrow B cell numbers in Cfh+/+ (blue dots) and Cfh−/− (red dots) mice quantified by flow cytometry. (B) Bar graphs representing the kappa/lambda light chain ratio of immature B cells in the bone marrow of Cfh+/+ (blue bar) and Cfh−/− (red bar) mice analyzed by flow cytometry. (C) Absolute numbers of mature bone marrow B cells and (D) their CD23 expression in Cfh+/+ (blue dots) and Cfh−/− (red dots) mice measured by flow cytometry. (E) Representative flow cytometry plots showing the gating strategy for circulating CD23+ B cells and dot plots indicating the count and (F) CD23 expression of CD23+ B cells in Cfh+/+ (blue dots) and Cfh−/− (red dots) mice quantified by flow cytometry. Data shown are pooled from two independent experiments. All results show mean ± SEM, each symbol represents an individual mouse, *p < 0.05, **p < 0.01 (unpaired t-test). [file Data_Sheet_1.zip › Figure 4.tif]

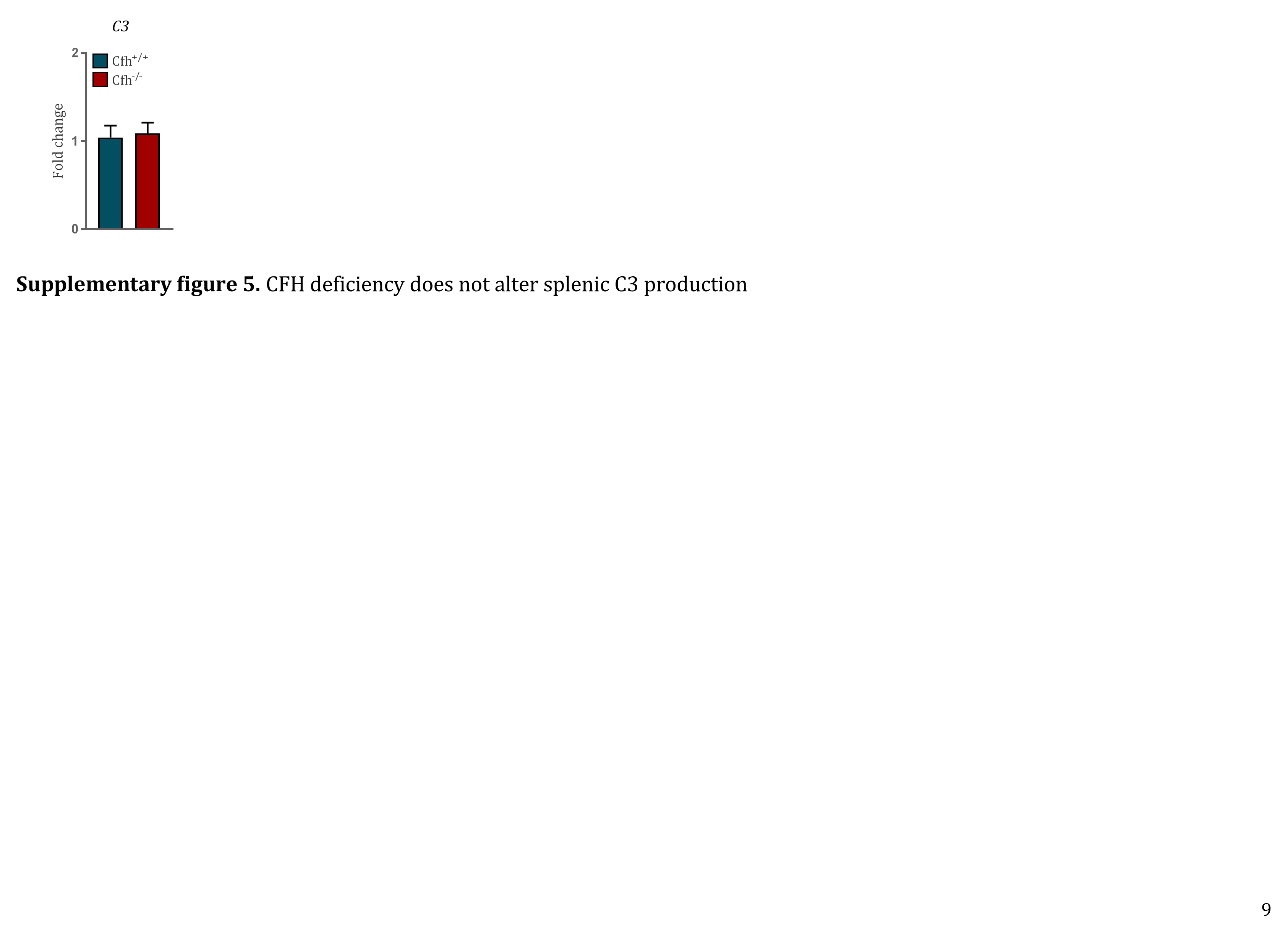

Supplement: Supplementary Figure 1 — CFH deficiency limits CD23 expression of circulating and bone marrow B cells without affecting their numbers. (A) Representative flow cytometry plots showing the gating strategy for B cells and dot plots demonstrating immature bone marrow B cell numbers in Cfh+/+ (blue dots) and Cfh−/− (red dots) mice quantified by flow cytometry. (B) Bar graphs representing the kappa/lambda light chain ratio of immature B cells in the bone marrow of Cfh+/+ (blue bar) and Cfh−/− (red bar) mice analyzed by flow cytometry. (C) Absolute numbers of mature bone marrow B cells and (D) their CD23 expression in Cfh+/+ (blue dots) and Cfh−/− (red dots) mice measured by flow cytometry. (E) Representative flow cytometry plots showing the gating strategy for circulating CD23+ B cells and dot plots indicating the count and (F) CD23 expression of CD23+ B cells in Cfh+/+ (blue dots) and Cfh−/− (red dots) mice quantified by flow cytometry. Data shown are pooled from two independent experiments. All results show mean ± SEM, each symbol represents an individual mouse, *p < 0.05, **p < 0.01 (unpaired t-test). [file Data_Sheet_1.zip › Figure 5.TIF]

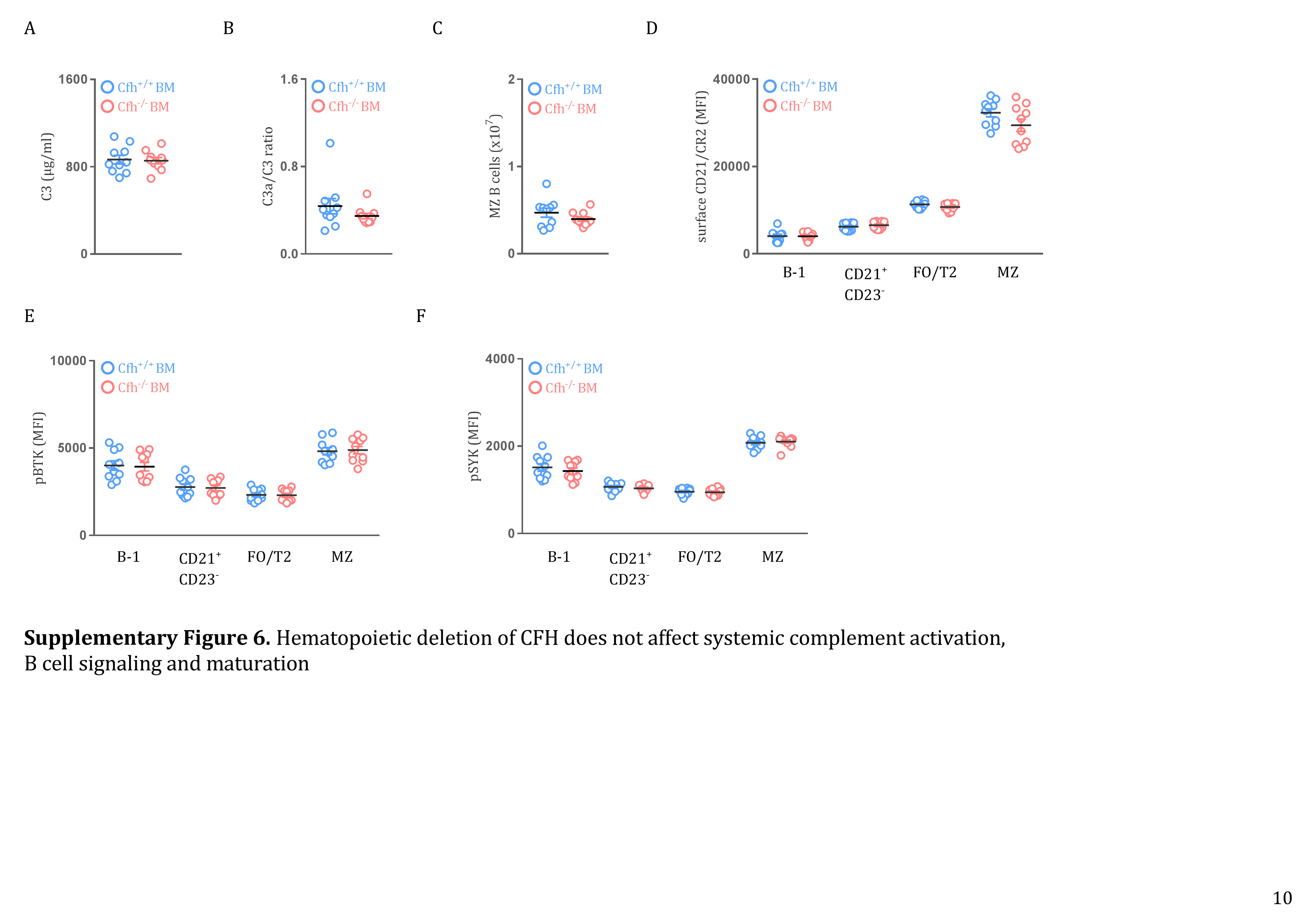

Supplement: Supplementary Figure 1 — CFH deficiency limits CD23 expression of circulating and bone marrow B cells without affecting their numbers. (A) Representative flow cytometry plots showing the gating strategy for B cells and dot plots demonstrating immature bone marrow B cell numbers in Cfh+/+ (blue dots) and Cfh−/− (red dots) mice quantified by flow cytometry. (B) Bar graphs representing the kappa/lambda light chain ratio of immature B cells in the bone marrow of Cfh+/+ (blue bar) and Cfh−/− (red bar) mice analyzed by flow cytometry. (C) Absolute numbers of mature bone marrow B cells and (D) their CD23 expression in Cfh+/+ (blue dots) and Cfh−/− (red dots) mice measured by flow cytometry. (E) Representative flow cytometry plots showing the gating strategy for circulating CD23+ B cells and dot plots indicating the count and (F) CD23 expression of CD23+ B cells in Cfh+/+ (blue dots) and Cfh−/− (red dots) mice quantified by flow cytometry. Data shown are pooled from two independent experiments. All results show mean ± SEM, each symbol represents an individual mouse, *p < 0.05, **p < 0.01 (unpaired t-test). [file Data_Sheet_1.zip › Figure 6.TIF]

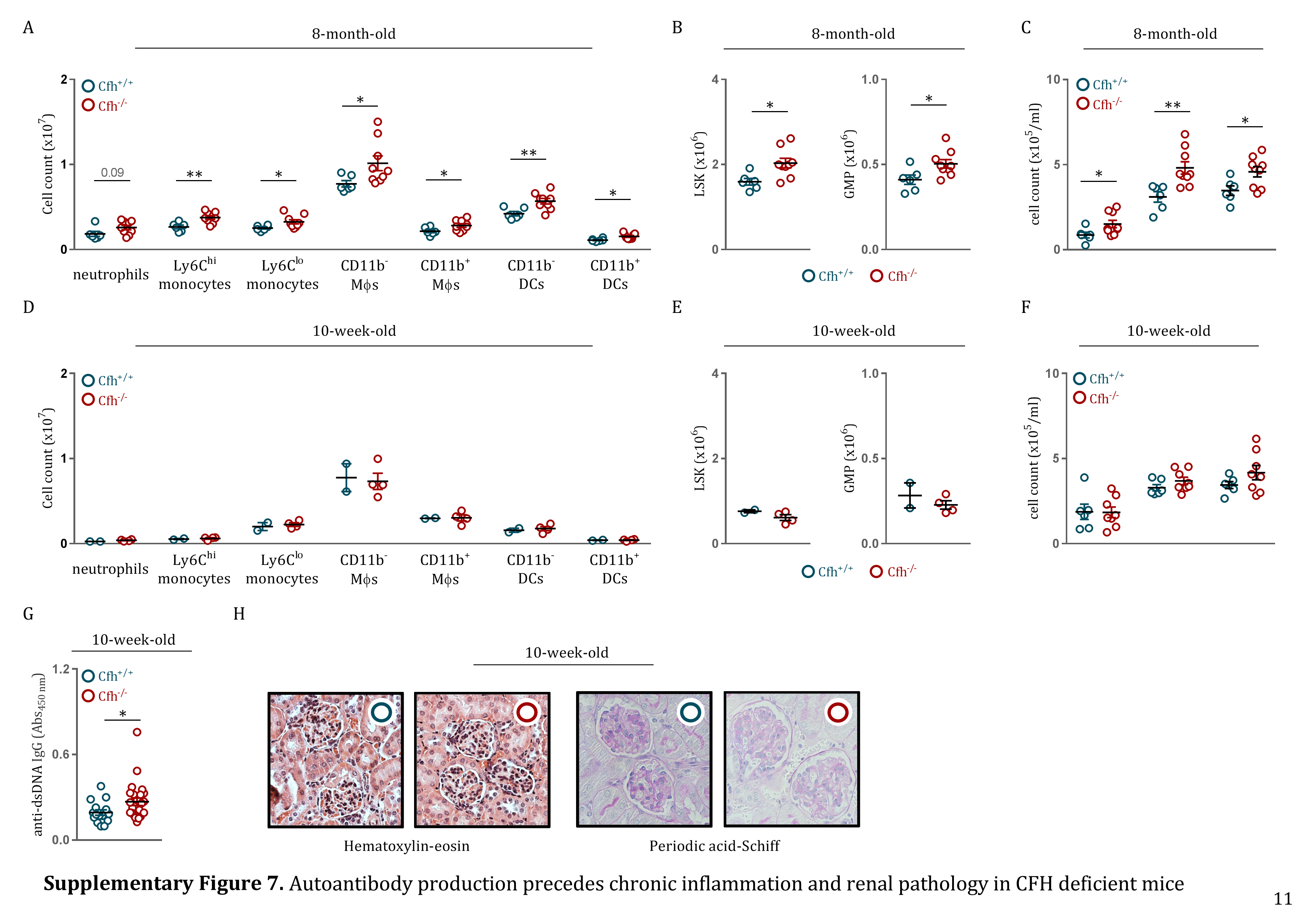

Supplement: Supplementary Figure 1 — CFH deficiency limits CD23 expression of circulating and bone marrow B cells without affecting their numbers. (A) Representative flow cytometry plots showing the gating strategy for B cells and dot plots demonstrating immature bone marrow B cell numbers in Cfh+/+ (blue dots) and Cfh−/− (red dots) mice quantified by flow cytometry. (B) Bar graphs representing the kappa/lambda light chain ratio of immature B cells in the bone marrow of Cfh+/+ (blue bar) and Cfh−/− (red bar) mice analyzed by flow cytometry. (C) Absolute numbers of mature bone marrow B cells and (D) their CD23 expression in Cfh+/+ (blue dots) and Cfh−/− (red dots) mice measured by flow cytometry. (E) Representative flow cytometry plots showing the gating strategy for circulating CD23+ B cells and dot plots indicating the count and (F) CD23 expression of CD23+ B cells in Cfh+/+ (blue dots) and Cfh−/− (red dots) mice quantified by flow cytometry. Data shown are pooled from two independent experiments. All results show mean ± SEM, each symbol represents an individual mouse, *p < 0.05, **p < 0.01 (unpaired t-test). [file Data_Sheet_1.zip › Figure 7.TIF]

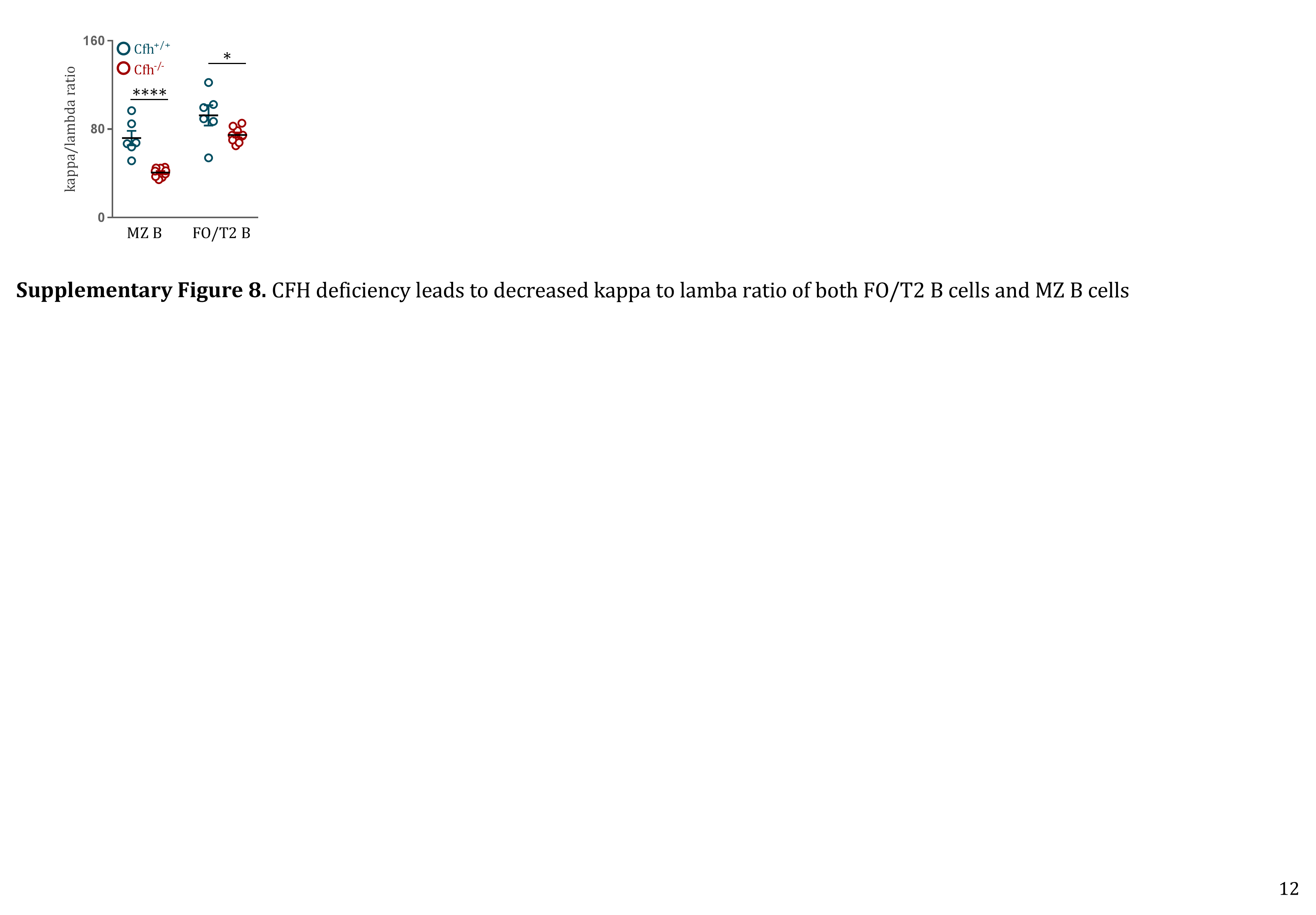

Supplement: Supplementary Figure 1 — CFH deficiency limits CD23 expression of circulating and bone marrow B cells without affecting their numbers. (A) Representative flow cytometry plots showing the gating strategy for B cells and dot plots demonstrating immature bone marrow B cell numbers in Cfh+/+ (blue dots) and Cfh−/− (red dots) mice quantified by flow cytometry. (B) Bar graphs representing the kappa/lambda light chain ratio of immature B cells in the bone marrow of Cfh+/+ (blue bar) and Cfh−/− (red bar) mice analyzed by flow cytometry. (C) Absolute numbers of mature bone marrow B cells and (D) their CD23 expression in Cfh+/+ (blue dots) and Cfh−/− (red dots) mice measured by flow cytometry. (E) Representative flow cytometry plots showing the gating strategy for circulating CD23+ B cells and dot plots indicating the count and (F) CD23 expression of CD23+ B cells in Cfh+/+ (blue dots) and Cfh−/− (red dots) mice quantified by flow cytometry. Data shown are pooled from two independent experiments. All results show mean ± SEM, each symbol represents an individual mouse, *p < 0.05, **p < 0.01 (unpaired t-test). [file Data_Sheet_1.zip › Figure 8.TIF]

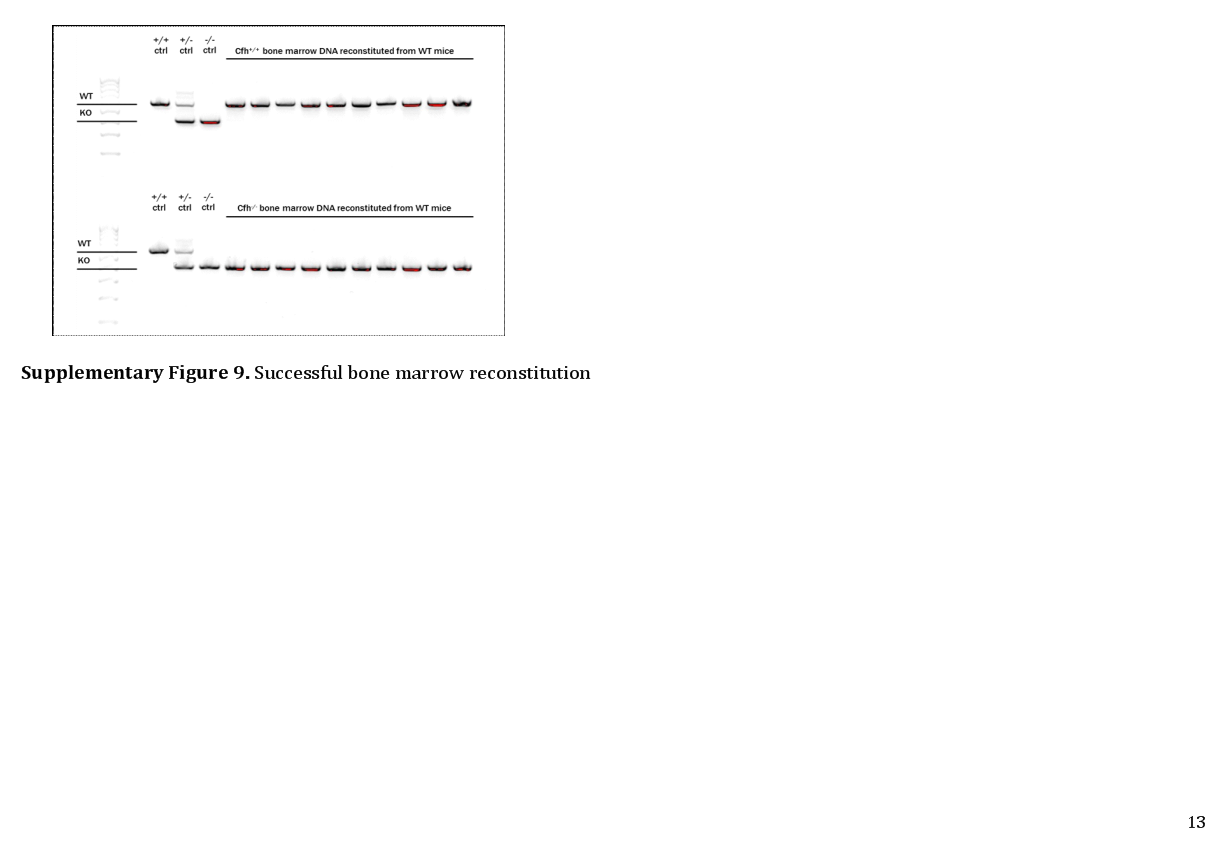

Supplement: Supplementary Figure 1 — CFH deficiency limits CD23 expression of circulating and bone marrow B cells without affecting their numbers. (A) Representative flow cytometry plots showing the gating strategy for B cells and dot plots demonstrating immature bone marrow B cell numbers in Cfh+/+ (blue dots) and Cfh−/− (red dots) mice quantified by flow cytometry. (B) Bar graphs representing the kappa/lambda light chain ratio of immature B cells in the bone marrow of Cfh+/+ (blue bar) and Cfh−/− (red bar) mice analyzed by flow cytometry. (C) Absolute numbers of mature bone marrow B cells and (D) their CD23 expression in Cfh+/+ (blue dots) and Cfh−/− (red dots) mice measured by flow cytometry. (E) Representative flow cytometry plots showing the gating strategy for circulating CD23+ B cells and dot plots indicating the count and (F) CD23 expression of CD23+ B cells in Cfh+/+ (blue dots) and Cfh−/− (red dots) mice quantified by flow cytometry. Data shown are pooled from two independent experiments. All results show mean ± SEM, each symbol represents an individual mouse, *p < 0.05, **p < 0.01 (unpaired t-test). [file Data_Sheet_1.zip › Figure 9.TIF]
